# Predictors of Major Depressive Disorder in Older People

**DOI:** 10.3390/ijerph182211894

**Published:** 2021-11-12

**Authors:** Susana Sousa, Constança Paúl, Laetitia Teixeira

**Affiliations:** 1 Institute of Biomedical Sciences Abel Salazar, University of Porto (ICBAS-UP), 4050-313 Porto, Portugal; paul@icbas.up.pt (C.P.); laetitiateixeir@gmail.com (L.T.); 2 Center for Health Technology and Services Research (CINTESIS), 4200-450 Porto, Portugal

**Keywords:** major depressive disorder, predictors, elderly, health

## Abstract

Major depressive disorder (MDD) is one of the most common mental disorders in older people. There are several biological, psychological, and social factors associated with this disorder. This study aimed to describe the depressive state to identify the associated factors and potential predictors of MDD in a population of community-dwelling older people with probable MDD. The sample consisted of 378 participants with probable dementia, with 47.3% of them presenting MDD. The factors that were found to be associated with MDD were sex, living status, mobility, and nutritional status. Knowing the factors that can predict a condition such as MDD is extremely important, both for prevention and for the customization of interventions.

## 1. Introduction

Mental health disorders are associated with reduced functional capacity and quality of life in the elderly. Major depressive disorder (MDD) is one of the most common mental health disorders in older people [1,2]. The estimated number of people with MDD worldwide in 2015 was 300 million [3]. In Portugal, the prevalence of MDD in the population over 65 years old is 7.5–12.6% [4].

MDD is a clinical condition characterised by a feeling of sadness and loss of interest in activities that were once perceived as enjoyable. To be considered a disorder, rather than just a normal reaction to life events, these symptoms must persist for at least 2 weeks and must be generally accompanied by changes in appetite and sleep patterns, fatigue, difficulty concentrating, indecision, suicidal thoughts, or feelings of worthlessness, helplessness, and despair [5].

MDD in older people involves the psychological, biological and social domains. There are several associated factors within these three domains that may have started in an earlier stage of life or that may be directly related to the ageing process. These factors may pertain to pathologies or health-related changes such as reduced physical strength, sensory acuity and information-processing speed and social changes such as retirement, widowhood, and loneliness. These changes may lead to the physical and social conditions present in depressive disorders.

MDD in older people is often undervalued and not treated due to its non-specific symptoms, or because it is confused with other comorbidities, such as cardiac pathologies, diabetes mellitus, malignant neoplasms, infections, and major neurocognitive disorders [6]. All these conditions may contribute to the onset of MDD in older people, and this disorder also acts in a bidirectional manner on the aforementioned diseases.

In many cases, MDD coexists with other comorbidities (major neurocognitive disorder (MND), etc.) in the same individual. Therefore, differential diagnosis is essential. It also needs to be clarified if MDD is a risk factor for MND, or if it is a phase that may trigger this disease. Conversely, MND may be a risk factor for MDD due to the patient’s behavioural and emotional reactions to MND [7].

The presence of depressive symptoms that do not yet meet the diagnostic criteria for MDD is nonetheless an indicator of the risk of developing MDD. As such, and to prevent MDD, it is essential to recognise its predictors, give them individual attention, and not regard them as merely consequences of other comorbidities [8]. In addition, when determining the presence of these factors in the older population, it is essential to integrate a biopsychosocial perspective to avoid the negative stereotyping of ageing and elderly people as sad and normally depressed.

Within the scope of the literature review in this study, the predictors of MDD can be grouped into sociodemographic, behavioural, health, and life event predictors. The sociodemographic predictors include the following: (i) sex (MDD is more common in women than in men, and this difference persists into old age for many reasons; feelings of loneliness and a low self-perception of health are common among depressed women [9]); (ii) age (in most of the previous studies, the increased presence of MDD and depressive symptoms was found in older age groups [10]); (iii) education level (individuals with low education levels have a higher risk of developing depressive symptoms; a higher education level is a resource for individuals when faced with stressful situations); (iv) marital status (there are significantly more cases of MDD among divorced and widowed individuals than among married individuals [11]; stressful events such as divorce, or the loss of family members and friends, may predispose one to a depressive state [12]); and (v) social support, both formal and informal (a multidimensional concept referring to the material and psychological resources to which people have access through their social networks [13]; the maintenance of social networks in the elderly provides them with psychological and social well-being [14]; low social support can be considered a risk factor for an individual’s health [15]).

As for the behavioural predictors of MDD, they include (i) nutritional status (we must consider the bidirectionality of nutritional status and MDD, as MDD can interfere with nutritional status, debilitating the elderly or making them malnourished, and the debilitated/malnourished state of the elderly can lead to MDD—in many studies, MDD has been correlated with weight loss [16]) and (ii) physical exercise (many studies have provided empirical evidence of the relationship between physical exercise and MDD in the elderly; in one study [17], the MDD levels of older people before and after their participation in the Portuguese National Walking and Running Programme, were examined with a 6-month interval and the depressive symptoms showed significant improvements from the pre- to the post-test. In another study [18], the MDD levels of older people who did various types of physical exercise were assessed, and it was found that the exercises reduced their MDD levels).

For the health predictors of MDD, they include (i) chronic diseases such as cardiovascular diseases, diabetes, and hypertension (several studies have shown that chronic disease is the factor most associated with MDD in older people [19], and that quality of life is compromised by the number of chronic diseases that an elderly person has, contributing to MDD; conversely, MDD also often worsens the clinical condition) and (ii) cognition (several studies have shown that functional capacity is positively correlated with an individual’s cognitive impairment [20]; older people who show greater cognitive decline also show greater functional impairment [21], and consequently, isolation and MDD).

Finally, with regard to the life event predictors of MDD, the literature has suggested isolation and the consequent emotional state of loneliness as common denominators, generating a bidirectional effect to MDD which may complicate research and make it difficult to determine their cause–effect relationships of MDD [14].

With this study, we establish the following objectives: (i) to describe the MDD state and identify its associated factors in a community-dwelling elderly population with probable MND and (ii) to identify the potential predictors of MDD in the same population.

## 2. Materials and Methods

### 2.1. Framework

This observational and cross-sectional study was part of a larger study called “Needs for Care of People with Dementia”. The study was submitted to the Ethics Committee of the Regional Health Administration of the North (Administração Regional de Saúde do Norte [ARSN]) and was unanimously approved on 7 January 2014 (Advice nº. 6/2014). More information on this study can be found in [22].

The study population consisted of Portuguese nationals, and the following were the study’s participant inclusion criteria: (i) user of a primary healthcare unit integrated in a Health Centre Grouping (ACES) of the area covered by ARSN and (ii) aged 65 years or more. On the other hand, the following were the study’s participant exclusion criteria: (i) user of a primary healthcare unit integrated in an ACES of an area not covered by ARSN; (ii) resident of a nursing home, or patient in a hospital or psychiatric institution; (iii) aged below 65 years; and (iv) without subjective complaints of memory deficits corresponding to stage 1 in the Global Deterioration Scale (GDS).

The sampling was carried out in four steps. In step 1, information on the population was obtained by sex and age group, which met the inclusion criteria based on the estimates published by the National Statistics Institute for 2012 (636,826 inhabitants aged 65 or more living in the geographical area covered by ARSN). In step 2, the number of people aged 65 or older with probable MND was estimated and stratified by age group on the basis of the data from the World Health Organization Dementia Report [23]. In step 3, the final sample size was calculated considering 1% of the distribution obtained in the previous step. Finally, in step 4, the distribution of the final sample by ACES, sex, and age group was determined. The sampling method that was used was the stratified probability sampling method.

### 2.2. Instruments

The variables that were studied were operationalised using the instruments cited in Table 1.

The participants’ characteristics were obtained using absolute and relative frequencies (for the qualitative variables) or mean and standard deviation (for the quantitative variables). The associations between the sociodemographic, health, behavioural factors, and MDD (yes/no), were assessed through the chi-squared test (for the qualitative variables), or the independent samples t-test (for the quantitative variables). On the basis of the previous results, and considering only the factors associated with MDD, a multivariable logistic regression model was carried out, considering the forward stepwise method for selecting the variables to be included in the final model. The sex and age variables were included as covariates in the final model, and the odds ratio (OR) and the 95% confidence interval (CI) were reported. Significance was set at 0.05 for all the analyses, and all the analyses were performed using the Statistical Package for the Social Sciences (SPSS) version 26.0 software (IBM Corp., Released 2019. IBM SPSS Statistics for Windows, Version 26.0. IBM Corp., Armonk, NY, USA).

## 3. Results

### 3.1. Sample Characteristics

Of the sample consisting of 378 participants with probable MND, over 50% were female and the overall mean age was 74.7 years (standard deviation [SD] = 7.1; range: 65–98 years). The mean years of education was 3.35 years (SD = 2.39; range: 0–17 years), with 17.8% of individuals having no formal education. As for marital status, most were married (61.3%), 16.8% were living alone, and 52.8% were living in an urban setting. Almost 30% had informal care support while 23.7% had formal care support (Table 2).

On the basis of the ABCDS instrument, the mean MDD score was 2.46 (SD = 1.77), with 47.3% of the participants classified as having probable MDD (ABCDS score ≥ 3).

### 3.2. Factors Associated with Major Depressive Disorder

Table 3 presents the study findings regarding the associations between MDD (yes/no) and the sociodemographic, health, and behavioural factors. For the sociodemographic factors, sex, years of education, marital status, and living status were found to be associated with MDD. More female participants, and participants with a lower level of education, with no partner (single, divorced, or widowed) and who were living alone, had MDD, than male participants, and participants with higher education, with a partner (married) and who were not living alone, respectively. For the health factors, only handgrip strength and mobility (TUG) were associated with MDD. Finally, nutritional status (MNA) and physical activity were the only behavioural factors associated with MDD.

### 3.3. Predictive Factors of Major Depressive Disorder

**Figure 1:**
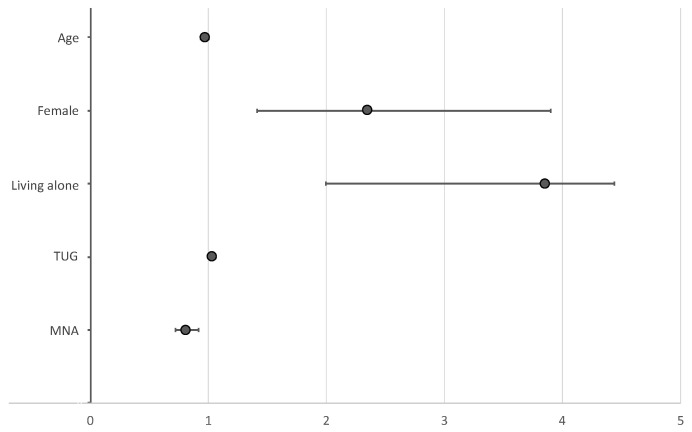
Predictors of major depressive disorder (odds ratio [OR] and 95% confidence interval [CI]).

Figure 1 shows the predictive factors of MDD that emerged from the multivariable analysis. Sex, living status, mobility (TUG), and nutritional status (MNA), were the predictive factors of MDD that were identified after adjusting for age and sex. Women (OR = 2.351; 95% CI = 1.417–3.900) and the elderly living alone (OR = 3.851; 95% CI = 1.994–7.436) were found to have a higher chance of acquiring MDD after adjusting for the remaining predictors. In addition, lower mobility (longer time to complete the TUG test; OR = 1.034; 95% CI = 1.006–1.062) and worse nutritional status (lower MNA score; OR = 0.814; 95% CI = 0.721–0.919) were found to be significant predictors of MDD after adjusting for the other factors.

## 4. Discussion

MDD is one of the most frequently observed mental disorders in the elderly [5] and has a major impact on both the patient and the caregiver, compromising their quality of life. It may even be a risk factor for many other health conditions, including MND [28]. The elderly experience frequent losses of family, social and economic support (retirement, greater physical decline, more physical illnesses, disability, etc.), often leading to isolation and loneliness, which may trigger MDD. However, MDD in older people is seldom recognised [29], and depressive symptoms are often attributed to physiological causes, or regarded as being associated with other comorbidities.

With regard to this study’s first objective, we found a 47.3% prevalence of MDD in an elderly population with probable MND, which is significant compared to the prevalence of MDD in the general population (7.5–12.6%) This difference could be explained by the specificity of the population that had probable MND, and the association between MND and MDD. Determining this and understanding the factors associated with MDD in the elderly are crucial so that a diagnosis can be made on the basis of the MDD screening results, and so that the cases can be appropriately treated and followed up.

We highlighted several factors in the study sample that proved to be indicators of MDD in the aforementioned study population. The female sex was found to be an indicator, closely associated with biological causes, such as hormonal changes, but also with social factors and roles that are similarly very relevant in elderly women (retirement, concern with their children and/or grandchildren, etc.) [30]. Another indicator of MDD is a low level of education, perhaps correlating with fewer chances to engage in leisure activities that may provide relief in stressful situations, eventually resulting in less autonomy in the search for social responses, and subsequently leading to greater isolation from activities. Not having a partner and living alone were also found to trigger MDD, probably due to social isolation. Weak handgrip, low mobility, poor nutritional condition, and low physical activity (and mobility), are health conditions that may configure frailty, thus posing a greater risk of isolation and triggering MDD [31].

The factors identified as relevant for predicting MDD in this study are in line with those identified in other studies [9,10,11,12,13,14,15].

All the aforementioned indicators of MDD, especially sex, living status, mobility, and nutritional status, seem to have a common denominator: isolation. In other words, these factors, in different ways, lead the elderly to a state of social isolation, leading to loneliness which may trigger MDD. Loneliness can be addressed through customised psychological intervention. Other aspects that should be addressed in interventions with the elderly are nutrition, mainly alimentation as a social activity, together with physical activity, which is also associated with significant outdoor group arrangements.

In the last two years, COVID-19 has added to the triggering factors of MDD. The effects of the disease, characterised by an excess of mortality in the older population, but also by the decrease and/or suspension of care by services, and by the families themselves, have seen an increase in depression of about 50% in people over 50 years of age [32]. Furthermore, the consequences of social distancing, one of the main measures of control of the epidemic, were negative in terms of mental well-being, especially for the elderly. In other words, there was an aggravation of social isolation [33].

Some limitations could be identified in this study. Firstly, related to the exploratory design of this study, MDD status was obtained with a brief screening instrument (ABCDS) and not a diagnosis, which may result in some bias in the estimation of the prevalence of MDD. Additionally, other relevant factors identified in other studies [34,35] were not considered (life events, such as divorce and the death of relatives).

Finally, the inclusion of a control group of individuals with MDD but no MND could have been relevant to compare prevalence of MDD in both groups.

This study showed that the importance of knowing the factors that can predict a condition such as MDD is extremely important both for the prevention of the condition and for the adoption of interventions for it.

## Figures and Tables

**Table 1:** Instruments included in the assessment protocol.

Instrument	Description
Sociodemographic questionnaire	Sex (male/female), age, number of years of education, social isolation (lives alone/does not live alone).
Global Deterioration Scale–GDS [24]	Qualitatively classify the individuals according to the stage of primary degenerative dementia. An overall description of the symptoms and clinical characteristics expected for each stage of dementia is provided in the instrument, and such descriptions are considered when deciding on the most appropriate global level (stage) of cognition and function.
AB Clinician Depression Screen–ABCDS [25]	Brief version of the Geriatric Depression Scale for clinical use. Five questions with two response options: yes and no. The final score of the scale ranges from 0 to 5, with the respondents with a score equal to, or higher than, 3 showing a high probability of depression.
Older Americans Resources and Services–OARS [26]	Assess functional capacity in five areas fundamental to the quality of life of the elderly. List of the most frequent problems of older people (e.g., cardiovascular problems, hypertension, diabetes and hearing loss).
Timed “Up and Go”–TUG [27]	Evaluates the functional mobility of the elderly. In the test, the individual is observed and timed while getting up from an armchair, walking 3 metres, returning, and sitting down again.
Short- Form Mini Nutritional Assessment-MNA-SF [28]	Nutritional-status screening instrument. Composed of six indicators: decreased food intake, weight loss, mobility, psychological stress or acute illness, neuropsychological problems, and body mass index. The score ranges from 0 to 14, with a higher score indicating a worse nutritional status.
Handgrip strength	Evaluates handgrip strength using a dynamometer. Four attempts are made, two on each hand, alternately. The final score is the average of the highest values obtained.
Physical-activity question	Assessing the frequency of one’s physical activity, such as gardening, house/car cleaning, or walking. Isolated question using a 4-point scale, where 1 = more than once a week, 2 = once a week, 3 = one to three times a month and 4 = almost never or never. These points are later aggregated into only two classes: 1 = regularly (more than once a week), and 2 = almost never or never (once a week, one to three times a month, and almost never or never).

**Table 2:** Sociodemographic characteristics of the sample.

	N (%) or Mean (SD)
Total	378
Sex (female)	222 (58.7)
Age (years), mean (standard deviation [SD])	74.7 (7.1)
Years of education, mean (SD)	3.35 (2.39)
Marital status	
Single	19 (4.9)
Married	231 (61.3)
Divorced/separated	19 (5.2)
Widowed	108 (28.6)
Living alone (yes)	63 (16.8)
Context (urban)	190 (52.8)
Informal care support (yes)	150 (29.8)
Formal care support (yes)	90 (23.7)

**Table 3:** Association between major depressive disorder and sociodemographic, health, and behavioural factors.

	Depression		*p*
	No	Yes	
Sociodemographic factors			
Sex			<0.001
Male	107 (68.6)	49 (31.4)	
Female	92 (41.4)	130 (58.6)	
Age (years), mean (SD)	74.8 (6.9)	74.7 (7.3)	0.903
Years of education, mean (SD)	3.68 (2.36)	2.97 (2.38)	0.004
Marital status			<0.001
Single	7 (38.9)	11 (61.1)	
Married	145 (62.8)	86 (37.2)	
Divorced/separated	8 (40.0)	12 (60.0)	
Widowed	38 (35.5)	69 (64.5)	
Living alone			<0.001
Yes	17 (27.0)	46 (73.0)	
No	181 (58.2)	130 (41.8)	
Context			0.428
Rural	93 (54.7)	77 (45.3)	
Urban	96 (50.5)	94 (49.5)	
Informal care support			0.130
Yes	72 (48.0)	78 (52.0)	
No	127 (55.9)	100 (44.1)	
Formal care support			0.226
Yes	42 (47.2)	47 (52.8)	
No	157 (54.5)	131 (45.5)	
Health factors			
High blood pressure			0.569
Yes	82 (51.6)	77 (48.4)	
No	27 (56.3)	21 (43.8)	
Cardiovascular diseases			0.189
Yes	51 (61.4)	32 (38.6)	
No	55 (51.9)	51 (48.1)	
Diabetes			0.197
Yes	47 (58.0)	34 (42.0)	
No	58 (48.7)	61 (51.3)	
Cognitive decline (GDS)			0.055
Very mild/mild	166 (55.5)	133 (44.5)	
Moderate/moderately severe	26 (39.4)	40 (60.6)	
Severe/very severe	7 (58.3)	5 (41.7)	
Handgrip, mean (SD)	22.0 (9.3)	17.8 (8.9)	<0.001
TUG, mean (SD)	16.5 (10.0)	20.5 (11.8)	0.001
Behavioural factors			
MNA, mean (SD)	11.6 (2.1)	10.5 (2.4)	<0.001
Physical activity			0.012
Regularly	160 (56.3)	124 (43.7)	
Almost never/never	38 (41.3)	54 (58.7)	
